# Comparison of quality of life and causes of hospitalization between hemodialysis and peritoneal dialysis patients in China

**DOI:** 10.1186/1477-7525-5-49

**Published:** 2007-08-02

**Authors:** Ai-Hua Zhang, Li-Tao Cheng, Ning Zhu, Ling-Hua Sun, Tao Wang

**Affiliations:** 1 Division of Nephrology, Peking University Third Hospital, Beijing, People's Republic of China; 2 Division of Nephrology, Peking University First Hospital, Beijing, People's Republic of China

## Abstract

**Background:**

Hemodialysis (HD) and peritoneal dialysis (PD) are important renal replacement treatment in end stage renal disease (ESRD), but the comparison of quality of life (QOL) and causes of hospitalisation between the two modalities in China is lacking. In the present study, we compared the two modalities in a multi-center study.

**Subjects and methods:**

Six hundred and fifty four HD and 408 PD patients were investigated from 10 hospitals in China from Sept, 2004 to Jan, 2005. Among the HD patients, there were 360 males and 294 females with a mean age of 57.22 ± 12.49 years (18–88 y). Among PD patients, there were 165 males and 243 females, with a mean age of 61.59 ± 12.65 years (22–89 y). Health related 36 items short form questionnaires (SF-36) were used to assess the quality of life. Hospitalisation data were collected and analyzed.

**Results:**

SF-36 domains of Body Pain (BP), General Health (GH), Role-Emotional (RE), Social Functioning (SF), Vitality (VT) and Mental Health (MH) were all significantly higher in the PD patients as compared to the HD patients although there was no significant difference in Physical Functioning (PF) and Role-Physical (RP) between the two groups. The two most common causes of hospitalisation in HD patients were cardiovascular disease (39.8%) and pulmonary infection (21.3%), while they were infectious peritonitis (47.6%) and cardiovascular disease (31.9%) in PD patients. The ever hospitalised patients had lower SF-36 scores in the domains of PF, BP, GH, RE, SF, VT and MH as compared to those of non-hospitalised patients.

**Conclusion:**

Our study indicated that with the current practice in China, PD patients may enjoy better quality of life than their HD counterparts. Our results also showed that the most common cause of hospitalisation was cardiovascular disease in HD patients and peritonitis in PD patients.

## Background

Both hemodialysis (HD) and peritoneal dialysis (PD) are established renal replacement therapies to treat patients with end-stage renal disease (ESRD) worldwide. According to the data of Registry of Dialysis and Transplantation in 2005 in China, there were about 59,000 dialysis patients in this country and 90% of them were on HD. Although HD and PD are thought to provide similar benefits to ESRD patients, the actual large scale comparisons of quality of life (QOL) and causes of hospitalisation between the two modalities is currently lacking in China. In literatures, health related 36 items short form questionnaires (SF-36) were used increasingly to assess the QOL in the studies of ESRD patients [1-4]. So we employed SF-36 as a tool and investigated the QOL and causes of hospitalisation in HD and PD patients in China based on multi-center data in the present study.

## Subjects and Methods

### Data collection

Demographic and clinical data of 661 HD patients and 412 PD patients were derived from 10 big hospitals located in different cities in China from Sept, 2004 to Jan, 2005. Demographic information and clinical history were collected through review of medical records and self-reported SF-36 questionnaires. We categorized socio-demographic variables as follows: age, currently marriage status (yes/no), employment status (yes/no). Comorbidity was assessed by using Charlson Cormobidity Index, with higher score reflecting increased severity of disease[5-8].

### SF-36 questionnaire

The SF-36 questionnaires were the main tool to assess QOL in dialysis patients[1-4]. The SF-36 was finally evaluated in eight domains: Physical Functioning (PF,10 items), Role-Physical (RP,4 items), Body Pain (BP,2 items), General Health (GH,5 items), Role-Emotional (RE,3 items), Social Functioning (SF,2 items), Vitality (VT,4 items) and Mental Health (MH,5 items). The score for each domain range from 0 to 100 with higher scores indicates better quality of life [1-4].

### Hospitalisation

Retrospective hospitalisation data were collected for year 2004. Among all the patients, 757 patients had complete data and were enrolled for the hospitalisation analysis, but only 309 patients of them were defined hospitalisation, the other were defined non-hospitalisation for access-related hospitalisation (HD patients artery-venous fistula operation, PD catheter implantation) and transplanted-related hospitalisation were excluded. Hospital admissions for a variety of disorders were analyzed [12].

### Statistical analysis

Demographic and clinical data were described as mean ± SD for continuous variables. The differences in demographic data and SF-36 scores between PD and HD groups were analyzed by independent t tests for normally distributed variables or nonparametric tests for non-normally distributed variables. The effects of age, gender, diabetic status and Charlson index were adjusted by covariate analysis. To perform covariate analysis, the logarithmically transformed SF-36 scores were treated as dependent variables, while the modality of dialysis was treated as a fixed factor and the age, gender, diabetic status and Charlson index as covariates. P < 0.05 was considered as statistically significant. Statistical analyses were performed using SPSS 11.0 version.

## Results

### 1. Characteristics of the studied dialysis patients

Of the 1073 patients enrolled in the study, 1062 completed the SF-36 questionnaire (98.9% of responding rate). Table 1 showed relevant socio-demographic and clinical characteristics of the 1062 respondents. The results showed that the PD patients were elder than the hemodialysis patients (P = 0.000), and there were more female patients in PD patients than that in hemodialysis patients (59.6% vs. 45.0%, P = 0.000). Less than 10% of dialysis patients were employed (HD 3.0% vs. PD 7.4%, P = 0.219). The PD patients had lower Charlson index than HD patients (4.30 ± 0.58 vs. 4.78 ± 1.06, P = 0.000). The causes of ESRD were glomerlulonephritis (HD 46.0% and PD 47.8%), hypertensive nephropathy (HD 16.8% and PD 15.4%), diabetic nephropathy (15.1% in HD vs. 10.2% in PD, P = 0.024), drug-induced renal damage (HD 13.5% and PD 9.6%).

**Table 1:** Demographic data of the dialysis patients

	HD(N = 654)	Percentage	PD(N = 408)	Percentage
Characteristic				
Age				
<40	51	7.7%	26	6.3%
40–49	135	20.6%	34	8.3%
50–59	167	25.5%	111	27.2%
60–69	195	29.8%	121	29.7%
70–79	94	14.4%	96	23.5%
80+	12	1.8%	20	4.9%
Age(mean)	57.22 ± 12.49		61.59 ± 12.65	
Gender				
% female	294	45.0%	243	59.6%
Employment				
Employed	20	3.0%	30	7.4%
Marital status				
%current married	609	93.11%	390	95.58%
Charlson comorbidity Index	4.78 ± 1.06		4.30 ± 0.58	
Cause of ESRD				
Glomerulonephritis	301	46.0%	195	47.8%
Hypertensive nephropathy	110	16.8%	63	15.4%
Diabetic nephropathy	99	15.1%	42	10.2%
Drug-induced renal lesion	88	13.5%	39	9.6%
Unknown cause	45	6.9%	53	13.0%
Others	9	3.2%	16	4.0%

Abbreviations: ESRD, end-stage renal disease.

### 2. Comparisons of quality of life

The average scores for SF-36 domains of BP, GH, RE, SF, VT and MH were higher in PD patients than those in HD patients except PF and RP. This difference was still significant even after adjustment of age, gender, diabetic status and Charlson index by covariate analysis (Table 2). However, there were no significant differences between the diabetic patients and non-diabetic patients in terms of physical health and health dimensions, neither between the female and male patients (Table 3 and Table 4).

#### 3.   Analysis of the causes of hospitalisation in dialysis patients 

HD patients had higher hospitalisation as compared to PD patients (47.8%vs 29.7%, P=0.000). In HD patients, the two most common causes of hospitalisation were cardiovascular diseases and infection (especially pulmonary infection), while they were infection (especially infectious peritonitis) and cardiovascular diseases in PD patients (Table 5).   

**Table 2:** Comparison of SF-36 scores between PD and HD patients.

SF-36 Domain	HD (n = 654)	PD (n = 408)	Unadjusted P	Adjusted P
PF	45.07 ± 30.86	49.88 ± 30.63	0.065	0.098
RP	22.36 ± 45.61	26.41 ± 48.05	0.109	0.176
BP	43.02 ± 22.92	52.16 ± 26.21	0.000	0.000
GH	27.65 ± 18.82	36.75 ± 21.72	0.000	0.000
RE	42.36 ± 47.94	57.65 ± 48.06	0.000	0.000
SF	48.89 ± 25.20	56.71 ± 25.02	0.000	0.001
VT	36.67 ± 22.58	45.40 ± 24.30	0.000	0.006
MH	58.77 ± 23.56	68.08 ± 21.83	0.000	0.009

Abbreviations: PF, Physical Functioning; RP, Role-Physical; BP, Body Pain; GH, General Health; RE, Role-Emotional; SF, Social Functioning; VT, Vitality; MH, Mental Health.

**Table 3:** Comparison of quality of life between gender groups.

SF36 domain	Male (n = 525)	Female(n = 537)	P
PF	45.17 ± 30.17	46.90 ± 30.29	0.398
RP	23.76 ± 43.72	25.56 ± 44.46	0.182
BP	43.58 ± 23.93	45.83 ± 27.64	0.156
GH	30.40 ± 19.98	32.17 ± 21.01	0.215
RE	47.94 ± 48.32	48.48 ± 48.80	0.877
SF	51.19 ± 22.57	53.56 ± 26.28	0.069
VT	39.71 ± 23.56	40.41 ± 23.74	0.098
MH	62.68 ± 23.71	63.01 ± 23.08	0.433

Abbreviations: PF, Physical Functioning; RP, Role-Physical; BP, Body Pain; GH, General Health; RE, Role-Emotional; SF, Social Functioning; VT, Vitality; MH, Mental Health.

**Table 4:** Comparison of quality of life between diabetic and non-diabetic patients

SF-36 domain	Non-diabetic(n = 921)	Diabetic(n = 141)	P
PF	50.45 ± 28.09	44.19 ± 30.96	0.134
RP	28.37 ± 46.60	23.24 ± 46.57	0.117
BP	46.16 ± 24.72	49.10 ± 24.31	0.313
GH	31.17 ± 19.54	31.15 ± 20.60	0.846
RE	49.72 ± 47.16	47.93 ± 48.76	0.792
SF	52.74 ± 26.05	51.76 ± 25.32	0.598
VT	42.13 ± 24.83	39.71 ± 28.43	0.269
MH	62.16 ± 21.65	62.38 ± 28.63	0.846

Abbreviations: PF, Physical Functioning; RP, Role-Physical; BP, Body Pain; GH, General Health; RE, Role-Emotional; SF, Social Functioning; VT, Vitality; MH, Mental Health.

### 4. The comparison of SF-36 domains between hospitalised and non-hospitalised dialysis patients

Among the two major domains of the SF-36, the physical health dimension scores were also statistically different between the hospitalised patients and non-hospitalised patients, and the ever hospitalised patients had significant lower SF-36 domain scores (containing PF, BP, GH, RE, SF, VT, MH, see in Table 6).

**Table 6:** Comparison of SF 36 domains between hospitalisation and non-hospitalisation patients.

SF 36 domain	Hospitalisation N = 309	Non-hospitalisation N = 448	P
PF	41.35 ± 18.00	46.01 ± 19.02	0.03
RP	22.57 ± 42.52	24.94 ± 44.48	0.179
BP	42.66 ± 21.22	46.67 ± 22.34	0.009
GH	26.64 ± 20.32	32.89 ± 20.44	0.01
RE	41.89 ± 48.12	52.20 ± 49.28	0.001
SF	49.67 ± 26.17	53.69 ± 23.62	0.001
VT	38.85 ± 22.41	40.61 ± 20.25	0.03
MH	63.43 ± 23.68	64.18 ± 23.32	0.03

Abbreviations: PF, Physical Functioning; RP, Role-Physical ; BP, Body Pain ; GH, General Health; RE, Role-Emotional ; SF, Social Functioning; VT, Vitality; MH, Mental Health.

## Discussion

Previous study reported that there was no simple answer to the question of which dialysis modality could be expected to provide better quality of life [9,10]. Several studies suggested advantages for PD in some domains [11,12], and HD in others [3] or little difference between the two modalities[13,15].

The current study performed in China showed that PD patients reported better quality of life in mental health dimensions, GH (Combined mental health dimensions and physical health dimensions) and BP of physical health dimensions than HD patients except PF and RP if non-matched demographic data were not adjusted. This difference remained significant after adjustment of the patients' characteristics. It was interesting that we failed to demonstrate that diabetic status affected QOL, which was contrasted to some studies [2], but consisted with our previous investigations [14]. The exact cause for better QOL in Chinese PD patients is not clearly at present, but we believe that the following reasons should be considered. Firstly, the lower body mass index in Chinese patients may be translated as an adequate dialysis could be achieved with a relatively lower dialysis dose. Indeed, there are reports that the survival in Hong Kong Chinese PD patients is better than that in Caucasian patients in Western world, although the former have relatively lower Kt/V [16]. Secondly, it is reported that the micro-inflammation state, a predictor of cardiovascular event in dialysis patients [17], is lower in Asian patients than that in Western patients [18], which may be due to the difference in race and/or dietary habits.

Our current study also showed that the RP score in Chinese dialysis patients was low, consisting with the low employment rate (HD 3.2%, PD 7%) observed in this study. This might be due to the following reasons: Firstly, many Chinese patients do not accept the concept of timely dialysis until they suffer more and severer comorbidity; Secondly, the Chinese dialysis patients usually depend on the care of their family members (their children, spouse, sisters and brothers, parents, etc); Thirdly, Chinese dialysis patients are often not re-employed because of their end stage renal disease.

The current study showed that the cause of hospitalisation differed between PD and HD. In our HD patients, cardiovascular disease was most common cause and infection ranked the second. Moreover, majority of cause of hospitalisation were congestive heart failure in HD patients. Our results were consisted with some previous reports. Rayner [19] reported that cardiac disease was a common cause of death in chronic hemodialysis patients. A sub-analysis of the data on cardiac diseases in the Hemodialysis (HEMO) Study by Cheung[20] found that among the total of 1685 cardiac hospitalisations, angina and acute myocardial infarction accounted for 42.7% of all the hospitalisations. Allon M[21] reported infectious complication was common in hemodialysis patients and the frequency of a severe outcome varied greatly by infectious disease category, being highest for cardiac infections (95.6%) and infection of unknown source (68.4%), and lowest for urinary tract infections (35.5%) and access-related infections (43.8%).

For PD patients, although majority of Chinese PD patients have been using twin-bag system of Baxter Ltd since late 1990's, infectious disease especially peritonitis remained to be the most common cause of hospitalisation in our patients indicating further efforts are need to decreased the incidence of peritonitis in this patient population.

Kalantar[22] reported that prospective hospitalisations of hemodialysis patients correlated significantly with the SF-36 total score and its two main dimensions. Our retrospective hospitalised patients had worse quality of life than non-hospitalised patients, decreasing cormorbidities should thus be an effective way to improve the quality of life in dialysis patients.

In conclusion, although the present study is not a randomized controlled study and the selection of dialysis modality may have been biased in many aspects, our study indicated that with the current practice in China, PD patients may enjoyed better quality of life than their HD counterparts. Our results also showed that the most common cause of hospitalisation was cardiovascular disease in HD patients and peritonitis in PD patients.

**Table 5:** Cause of hospitalisation for dialysis patients (number of patients)

Cause of hospitalisation	HD	PD
Infection-related	47*#	41*#
Cardiovascular disease	89*#	30*#
Hemorrhage	21	
Vascular access	34	
PD technique problem		7
Others	32	8
Total	223	86#

* P < 0.01 Compared with within HD and PD patients by Chi-square test

# P < 0.01 Compared between HD and PD patients by Chi-square test
